# Investigation of utilization of nanosuspension formulation to enhance exposure of 1,3-dicyclohexylurea in rats: Preparation for PK/PD study via subcutaneous route of nanosuspension drug delivery

**DOI:** 10.1186/1556-276X-6-413

**Published:** 2011-06-07

**Authors:** Po-Chang Chiang, Yingqing Ran, Kang-Jye Chou, Yong Cui, Harvey Wong

**Affiliations:** 1 Small Molecule Research. Genentech, 1 DNA Way, South San Francisco, CA 94080, USA

## Abstract

1,3-Dicyclohexylurea (DCU), a potent soluble epoxide hydrolase (sEH) inhibitor has been reported to lower systemic blood pressure in spontaneously hypertensive rats. One limitation of continual administration of DCU for *in vivo *studies is the compound's poor oral bioavailability. This phenomenon is mainly attributed to its poor dissolution rate and low aqueous solubility. Previously, wet-milled DCU nanosuspension has been reported to enhance the bioavailability of DCU. However, the prosperities and limitations of wet-milled nanosuspension have not been fully evaluated. Furthermore, the oral pharmacokinetics of DCU in rodent are such that the use of DCU to understand PK/PD relationships of sEH inhibitors in preclinical efficacy model is less than ideal. In this study, the limitation of orally delivered DCU nanosuspension was assessed by a surface area sensitive absorption model and pharmacokinetic modeling. It was found that dosing DCU nanosuspension did not provide the desired plasma profile needed for PK/PD investigation. Based on the model and *in vivo *data, a subcutaneous route of delivery of nanosuspension of DCU was evaluated and demonstrated to be appropriate for future PK/PD studies.

## Introduction

In recent years, researchers have demonstrated that various epoxyeicosatrienoic acid (EETs) regioisomers cause either vasodilatation or vasoconstriction in a number of vascular beds [1-3] and that they hold anti-inflammatory properties [4]. There is compelling evidence from the literature that increasing the levels of EETs demonstrates anti-inflammatory, cardio-protective [5-8] antihypertensive, and renal vascular protective effects during disease states. These properties make this pathway an extremely attractive target for intervention. Based on these findings, soluble epoxide hydrolase (sEH) inhibition is a potentially attractive pharmacological approach to treat human hypertension. It has been reported that 1,3-dicyclohexyl urea (DCU) is a potent sEH inhibitor and inhibits human vascular smooth muscle (VSM) cell proliferation in a dose-dependent manner [9,10]. Because of the anti-inflammatory and antihypertensive properties of sEH inhibition, DCU can be used as a model sEH inhibitor to further investigate decreased VSM cell proliferation, a crucial pathologic mechanism in the progression from systemic hypertension to the atherosclerotic state [4,11,12]. However, despite having high *in vitro *potency, the utility of DCU to investigate sEH is limited based both on its short t_1/2 _in rats [13-15] and its low aqueous solubility, which makes oral delivery of DCU to maintain prolonged and constant exposure difficult. Such an issue is not DCU specific. It is well acknowledged in the pharmaceutical industry today that an increasing number of lipophilic drug candidates are providing scientists with the growing challenge of reaching desired exposures *in vivo*. Approaches to deliver poorly soluble molecules have been developed for both clinical and preclinical activities [14-17]. However, in the early phase of drug discovery where large numbers of potential candidates are screened, development of suitable formulations in time for a drug candidate's *in vivo *evaluation remains a big challenge. In general, formulations made at this early stage need to be prepared on a small scale using common excipients with little lead development time and the assurance of reliable delivery of target concentration levels.

Recently, nano- and microparticle drug delivery has been widely used in the pharmaceutical industry as a tool to overcome exposure issues [17-23]. Previously, much improved exposures were reported when nanosuspension formulations were used to deliver DCU [13-15]. Improvements in oral exposure by a DCU nanosuspension formulation enabled a dose-dependent efficacy study in a diseased animal model [14]. Despite the success of demonstrating preclincal efficacy, further utilization of DCU as a tool to evaluate target PK/PD relationships in chronic animal models [24] remains challenge. A short t_1/2 _coupled with a high drug plasma peak to trough (P/T) ratio was observed when DCU nanosuspension was dosed orally in rats [14].

In order to have full confidence of chemistry strategy for drug research, a full understanding of PK/PD relationships is essential when new targets are explored. The short apparent oral t_1/2 _(2.6 h) [14] and the high plasma P/T ratio limits the ability of dosing DCU nanosuspension orally to characterize PK/PD relationships in detail. In this case, the short t_1/2 _of DCU required twice daily (b.i.d.) to three times daily (t.i.d.) dosing to cover the target plasma IC50 and multiples. In addition, the high plasma P/T ratio confounds the researcher's ability to understand IC50 coverage requirements needed for *in vivo *efficacy. For example, it is very difficult to determine if the observed efficacy is driven by maximum plasma concentration (*C*_max_) or minimum plasma concentration (*C*_min_) when such a steep drop of DCU plasma exposure is encountered [14]. Unless full PK/PD relationships can be determined, the drug target candidate profile for first in class targets cannot be established with confidence; consequently, chemistry strategy cannot be implemented without risks.

In order to overcome this issue, the delivery of DCU via intravenous (IV) infusion route was explored. Similar to oral delivery, IV delivery of DCU was limited by the poor aqueous solubility of DCU. The poor aqueous solubility of DCU is such that it cannot be formulated for IV delivery without a high percentage of organic cosolvents which is incompatible with animal models in terms of efficacy. An alternative IV formulation using nanosuspension has also been evaluated in rats and demonstrated as a valuable option [13]. However, due to the complexity of the setup, such technique is only suitable for short term study (i.e., 2-4 h). The tool of delivering DCU to a chronic model for preclinical PK/PD still remains unanswered.

In this research, a drug surface area-based *in vivo *absorption model was established to evaluate the limitation of oral dosing DCU nanosuspension with respect to *in vivo *coverage. Due to the limitation of an inadequate t_1/2 _and a high plasma P/T ratio associated with oral dosing of DCU nanosuspension, it was concluded that an adequate and sustained coverage without a high plasma P/T ratio was not easily achievable by the oral route. In this investigation, a subcutaneous (SC) route of delivery of the nanosuspension of DCU was tested and was found to be suitable for future PK/PD studies. The findings confirmed our previous hypothesis and strongly support the use of SC dosing of DCU nanosuspension in the disease model (rat) to evaluate PK/PD relationships.

## Materials and methods

HPLC grade acetonitrile was obtained from Burdick & Jackson (Honeywell Burdick & Jackson, Muskegon, MI, USA), the reagent grade formic acid was obtained from EM Science (Omnisolve, EM Science, Gibbstown, NJ, USA), and 1,3-dicyclohexyl urea, Tween 80 were purchased from Sigma-Aldrich (Sigma-Aldrich Corp., St. Louis, MO, USA).

Lead-free glass beads (0.5-0.75 mm) were purchased from Glen Mill (Glen Mill's, Inc., Clifton, NJ, USA) and were preconditioned in-house. The water purification system used was a Millipore Milli-Q system (Millipore, Billerica, MA, USA). The XRPD pattern was recorded at room temperature with a Rigaku (Rigaku Americas Corp., The Woodlands, TX, USA) MiniFlex II desktop X-ray powder diffractometer. Radiation of Cu Kα at 30 kV-15 mA was used with a 2θ increment rate of 3°/min. The scans ran over a range of 2-40° 2θ with a step size of 0.02° and a step time of 2 s. The powder samples were placed on a flat silicon zero background sample holder. The particle size distribution of a regular suspension and nanosuspension was measured by using a Mictrotrac^® ^S3500 (Mictrotrac, Inc., Montgomeryville, PA, USA) instrument. Triplicates were measured for each sample, and the average was used for the final particle size distribution. The particle size distribution was calculated based on the general purpose (normal sensitivity) analysis model and the following refractive indices (RI): particle RI, 1.58; absorption, 1.0; and dispersant RI, 1.38.

## Formulation

For the particle size reduction, a bench scale wet milling devise was developed as described by Chiang et al. [13]. To prepare a nanosuspension stock formulation (50 mg/mL), bulk DCU, an appropriate amount of glass beads (1.5 times weight by weight of the final formulation), and a vehicle containing 0.5% (*w*/*w*) Tween 80 in phosphate saline (pH 7.4) were added in a scintillation vial to the desired volume. The mixture was then stirred on at 1,200 rpm for a period of 24 h with occasional shaking to prevent a buildup of the drug around the vial. The stock formulation was harvested by filtration to remove the glass beads. The same vehicle (0.5% (*w*/*w*) Tween 80 in phosphate saline pH 7.4) was used to prepare the regular suspension. For the regular suspension, a formulation was made by directly suspending bulk DCU in the vehicle. Formulation concentrations were verified by liquid chromatographic tandem mass spectrometric (LC/MS/MS).

**Figure 1:**
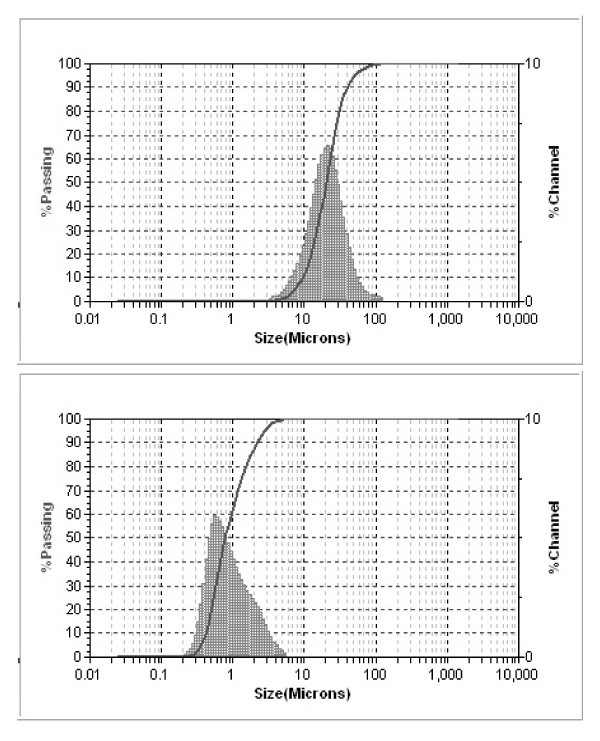
**DCU particle size analysis**. Regular suspension (top) and nanosuspension (bottom).

**Figure 2:**
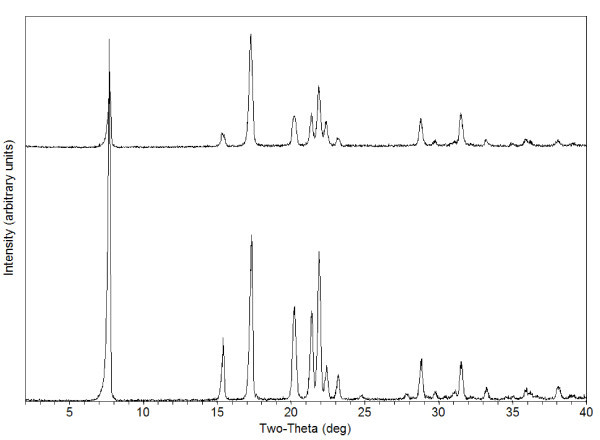
**PXRD DCU before milling (bottom) and post milling (top)**.

The stability of the DCU formulations (both regular suspension and nanosuspension) was assessed, and no issue was found. No particle size, potency, and form change was observed in a period of 7 days. All samples were found to be consistent with the previously reported data [13-15]. In general, an analysis of unmilled and milled DCU particles revealed a mean particle size of 20.2 μm (regular suspension) and 0.8 μm (nanosuspension), respectively (Figure 1). No form change was detected by PXRD when compare pre and post milling sample (Figure 2). The rate of dissolution of the DCU nanosuspension versus regular suspension is expected to increase at least 20-folds. To estimate the impact of dissolution, the Noyes and Whitney equation was used:

where d*C*/d*t *is the dissolution rate (*R*), *D *is the solute diffusion coefficient, *S *is the surface area of solute, *C*_s _is the saturation solubility of the solute, *C*_t_(*t*) is the bulk solute concentration, *V *is the volume of dissolution medium, and *h*_d _is the diffusion boundary thickness.

## Animals

Male Sprague-Dawley rats weighing between 290 and 350 g, obtained from Charles Rivers Laboratories (Charles Rivers Laboratories, Inc., Wilmington, MA, USA), were housed in a room with an ambient temperature of 22°C ± 1°C on a 12-h light/dark cycle. The animals were allowed 7 days to acclimate and were given *ad libitum *access to standard rat chow (0.5% NaCl) (Baxter Healthcare Corporation, Deerfield, IL, USA) and tap water until the initiation of the experiment [14]. The current study was conducted in accordance with the institutional guidelines for humane treatment of animals and was approved by the IACUC of Genentech. For dosing, each group of three male Sprague-Dawley rats was given either a 30-mg/kg subcutaneous dose of DCU formulated as regular suspension or nanosuspension. Oral dosing followed the same guidelines [14]. At the initiation of the study, the rats weighed from 297 to 329 g. Blood samples (approximately 0.2 mL per sample) were collected from each animal *via *jugular vein cannulae at the following time points: predose; 5, 15, and 30 min post dose; and 1, 2, 4, 8, and 24 h post dose. All samples were collected into tubes containing potassium ethylenediaminetetraacetic acid as an anticoagulant. Blood samples were centrifuged within 30 min of the collection, and plasma was harvested. Plasma samples were stored at approximately 70°C until analysis for DCU concentrations by a LC/MS/MS assay method.

## LC/MS/MS analysis

DCU plasma concentrations were quantified by using LC/MS/MS. Briefly, an internal standard (in-house compound) was added to samples followed by protein precipitation involving the addition of acetonitrile. Chromatography of DCU was achieved using a HALO Phenyl Hexyl column (2 × 50 mm, 2.7 μM particle size) (Advanced Materials Technology, Wilmington, DE, USA). The mobile phase used was 0.1% formic acid (A) and acetonitrile with 0.1% formic acid (B). A gradient was used and is described as follows: 10% B at 0 min and hold for 0.2 min, linear gradient to 95% B at 0.8 min and hold until 1.2 min, back to 10% B at 1.25 min and hold until 2.0 min. The total run time was 2.0 min, and the flow rate was 0.75 mL/min. An AB Sciex QTRAP 5500 mass spectrometer was used for detection. The MRM transition monitored for DCU was m/z 225.4 to m/z 100.2. The lower limit of quantitation was 0.013 μM (*S*/*N *= 6) in plasma.

## Dose simulation

A model based on the Wagner-Nelson (W-N) equation was established in-house and was used to calculate the drug absorbed to further assess the amount of drug absorbed as a function of time [25,26]. The utilization of the W-N equation allows us to obtain all the drug that is absorbed (including excreted) at different time points. This allowed us to estimate the relationship and the impact on the absorption on the surface area changes of the drug.

where *A *is the drug absorbed, *V *is the volume of distribution, Cp is the plasma concentration, *K *is the elimination rate constant, and *t *is time.

A slightly simplified gastro transit time equation was integrated in the model [27] to estimate the amount of drug entering the small intestine as a function of time.

where *M *is the mass of the drug remaining in the stomach, *D *is the drug dosed, Ke is the stomach empting rate, and *t *is the time.

A nonpsychological model was used to estimate the total available surface area of the DCU as a function of time. A linear movement was assumed in the GI [25,26].

## Result and discussion

The use of nanoparticles and particle size reduction in general to increase *in vivo *exposure for poorly soluble drugs is well practiced [17-23]. Reducing the particle size increases the surface area available to the dissolution media and thus increases the overall apparent drug dissolution. This can be estimated by the equation developed by Noyes and Whitney. Despite the understanding of surface area impact on the drug dissolution, the degree of impact on absorption by dosing nanoparticles remains unclear [25]. In theory, the best usage of utilizing nanoparticles to improve *in vivo *exposure (dissolution) is to dose it within the dissolution control range. In which the higher surface area of the nanoparticles is translated into a higher *in vivo *exposure.

An oral dose of DCU nanosuspension has been reported to greatly improve the *in vivo *exposure [14]. However, the overall limit of improvement that a nanosuspension formulation can provide for orally dosed DCU is not well understood [14]. In order to understand the degree of improvement provided by an oral nanosuspension formulation, simulations were performed using a Wagner-Nelson equation-based model that was established in-house in order to assess the amount of drug absorbed (d*A*) as a function of time [26,27]. In this model, the stomach empting time was taking into consideration. A log linear gastro transit model [28] was used to estimate the amount of drug available (*W*) in the small intestine for absorption. The surface area of the DCU was estimated by assuming a sphere shape particle and a true density (*d*) of 1.3 cm^3^/gm. The total surface area (*A*) was estimated by first obtaining the particle volume (*V*) using the equation of *V *= 3/4 *πr*^3^, and then total particle number (*n*) using the equation of *n *= ((drug weight)/*V*/*d*). The total surface area of the dose was estimated by the equation *A *= (4 *πr*^2^) × *n*. The unit surface area by weight (*A*/*W*) was calculated to estimate the surface area reduction after the absorption took place, and the total residual surface area (RA) was calculated for each time point. The absorption efficiency (AE) was calculated by taking the ratio of the amount of drug available and was divided by the RA (AE = *W*/RA), and the absorption constant (*K*) was calculated as AE/*δT*.

**Figure 3:**
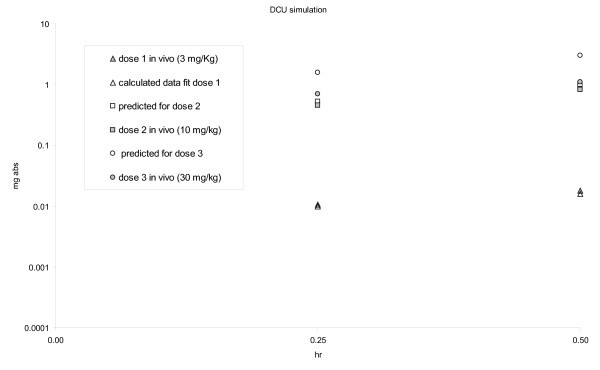
**DCU *in vivo *exposure model fit (SD rat PK)**.

All of the above parameters were obtained by using the 3-mg/kg rat oral PK data with regular suspension [14] as the base case and predictions were performed for higher doses (10 and 30 mg/kg) with nanosuspension formation. Results for 3, 10, and 30 mg/kg are listed in Table 1. According to theory, this model should hold within the linear range where absorption efficacy AE should be very close (amount of drug absorbed is affected by dissolution hence surface area) if oral absorption is dissolution rate-limited and should show deviations when absorption becomes solubility rate-limited. Within the linear range, an increased surface area (i.e., due to the nanolized drug) will result in a linear increase of oral absorption. This model was found to be sufficient to predict the exposure for dissolution rate-limited absorption at a 10-mg/kg dose. A much bigger deviation was observed at a 30-mg/kg dose when the predicted verse observed was compared with the absorbed amount (Figure 3). According to the model, at *C*_max_, a total of 3.0 mg of DCU should be absorbed where only 1.1 mg was observed *in vivo *(Table 1). A reduction in absorption efficiency (AE) was observed particularly between the 10- and 30-mg/kg doses (Table 1). These changes suggested that at a 30-mg/kg dose, the absorption is no longer dissolution rate-limited and most likely solubility rate-limited. The simulations suggest that doses of DCU that are higher than 30 mg/kg delivered using nanosuspension will not provide significantly higher exposure *in vivo*. Based on the modeling, doses higher than 30 mg/kg PO were not tested *in vivo*. Simulations for oral dosing were performed using the 30-mg/kg oral dose in order to assess the dose frequency required to hit a range of target concentrations.

**Table 1:** Predicted dug absorption (at *C*_max_) versus *in vivo *data (Wagner-Nelson equation) based on the surface area model

Dose/Drug absorbed in mg (impact by surface area only)	*In vivo *(Wagner-Nelson equation) mg	Predicted (mg)	**Total surface area of the drug dose (cm**^**2**^**)**	**Absorption efficiency (AE) mg/cm**^**2**^
3 mg/kg (regular suspension)	0.02	0.02	2.08 E0	9.0E-6
10 mg/kg (nano suspension)	0.84	1.01	1.38 E2	7.2 E-6
30 mg/kg (nano suspension)	1.11	3.03	4.15 E2	2.6E-6

**Figure 4:**
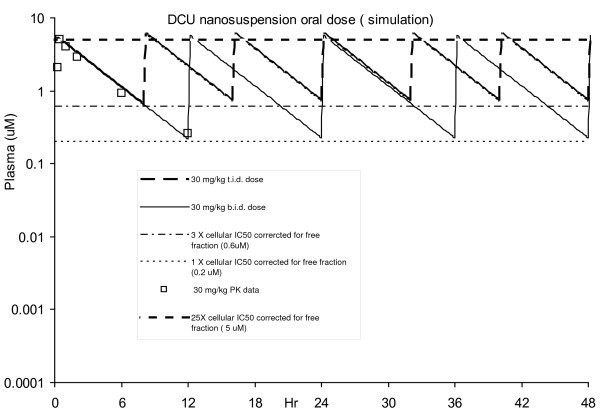
**DCU nanosuspension oral dose simulation for PK/PD**.

A prediction of the oral dose amount and frequency to cover different plasma concentrations were based on maintaining free fraction plasma concentrations of DCU (3% unbound) above a multiple of the cellular IC50 (6 nM) at the trough levels. Modeling of the pharmacokinetic data was performed using in-house model (1 compartment, first-order elimination), the pharmacokinetic parameters *V*_f _(5.8 L), *K*_01 _(absorption rate constant, 26.3 h^-1^) and *K*_10 _(elimination rate constant, 0.277 h^-1^) were estimated [15]. Several concentrations were used as "target coverage" since PK/PD investigations often require a broad range of target coverage (i.e., from 0.25 × IC50 to 10 × IC50). Based on the simulation (figure 4), oral dosing of 30 mg/kg of DCU nanosuspension twice a day (b.i.d.) is needed to provide continuous coverage of the plasma concentrations of 0.2 μM (1 × cellular IC50 corrected for free fraction) and t.i.d. dosing will be needed to cover 0.6 μM (3 × cellular IC50 corrected for free fraction). The increase in dosing frequency in order to cover three time the cellular IC50 is one shortcoming for the oral dosing of DCU especially for chronic studies. An additional drawback of this design is the high plasma P/T ratio. Higher than needed exposure resulting from the high P/T ratio can result in unwanted side effects and confound the efficacy read out [29]. Thus, oral dosing DCU to obtain the PK/PD relationship remains less than ideal.

**Figure 5:**
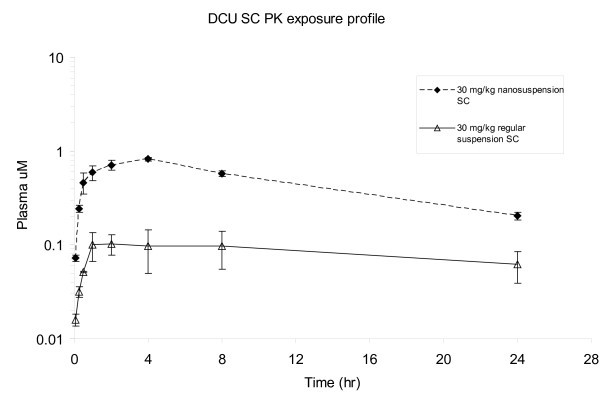
**DCU SC PK plasma exposure**. Thirty-milligram per kilogram nanosuspension versus regular suspension).

The SC route of delivery of the DCU nanosuspension in rats was investigated as a means to improve the pharmacokinetic properties of DCU. There are two potential benefits to investigate the SC dose for DCU. First, unlike oral absorption where all drug absorbed will first go through the liver then the circulation, the drug absorbed via the SC route will go directly into the circulation and hence avoid the "first pass" effect and potentially improve systemic exposure [30]. Secondly, the SC drug depot should continuously provide a slow release of drug to the bloodstream providing a longer and sometimes steady drug supply. Combined, both effects may result in a drug plasma profile with a more sustained drug coverage and lower P/T ratio. Despite the described advantages, the SC route of dosing is not free of problems. Drug exposure via SC route of delivery can be still limited by absorption, stability, dissolution rate, and solubility of the drug. In order to overcome these limitations, a suitable formulation was needed to maximize the potential of DCU *in vivo*. Several formulations strategies for SC dose have been assessed. Formulations such as emulsions and cosolvents were quickly found unsuitable since the goal was to target a formulation that can be directly applied to the efficacy model without any interference of excipients (i.e., high organic). After carefully evaluating all available options, nanosuspension was found to be the best option for the purpose. In order to understand the impact of nanosuspensions on the systemic exposure of DCU, both nanosuspension and regular suspension were dosed *in vivo *to contrast. It was found that when dosed via the SC route, the DCU nanosuspension greatly improved the exposure when compared with regular suspension. Results of this SC investigation are illustrated in Figure 5.

The SC dose of DCU with nanosuspension was very successfully. With DCU nanosuspension, an approximately threefold improvement of an apparent t_1/2 _was observed in SC dosing (10.2 h) in comparison to oral dosing (2.6 h). In addition, SC dosing resulted in a lower plasma P/T (*C*_max_/*C*_min 24 h_) ratio of 4. This much improved plasma P/T ratio was consistent with slower release. Furthermore, the nanosuspension was found to greatly improve the exposure and variability of the SC dose compared to the regular suspension. The regular suspension demonstrated similar effects on the exposure profile of DCU nanosuspension; however, a much reduced absorption rate (δc/δt) and lower exposure was observed (Figure 5). The exposure obtained via regular suspension at 30 mg/kg dosed was at least fivefold less when compared with the nanosuspension and results are listed as Table 2. It is hypothesized that the much reduced exposure was caused by the slower dissolution (dissolution rate-limited absorption) of the regular suspension which makes it unsuitable for a PK/PD study where higher exposures are needed.

**Table 2:** SC dose exposure comparison (nanosuspension versus regular suspension)

Dose (mg/kg)	***C***_**max **_**(μM) ± STDEV**	***C***_**min24hr **_**(μM) ± STDEV**	**AUC**_**0-t **_(h*μM) ± STDEV	**t**_**1/2 **_**(h) ± STDEV**
30 mg/kg (nanosuspension)	0.82 ± 0.03	0.20 ± 0.02	11.0 ± 0.5	10.2 ± 0.8
30 mg/kg (regular suspension)	0.13 ± 0.02	0.06 ± 0.02	2.0 ± 0.8	34.4 ± 21.4

**Figure 6:**
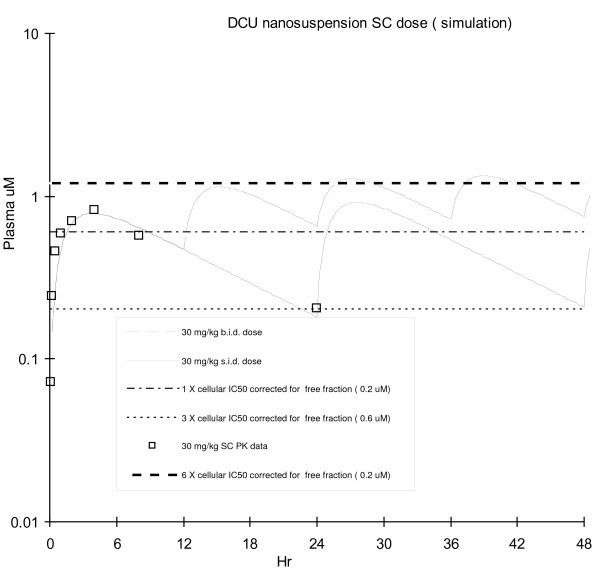
**DCU nanosuspension SC dose plasma exposure simulation**. Exposure versus cellular IC50 corrected for free fraction).

Modeling of the pharmacokinetic data was performed using the same in-house model (one compartment, first-order elimination) and revised to fit the *in vivo *data for SC dose. Based on the simulation, SC dosing of 30 mg/kg DCU nanosuspension once a day (s.i.d.) can provide continuous coverage of the plasma concentrations 0.2 μM (1 × cellular IC50 corrected for free fraction) and b.i.d. dose will cover 0.6 μM (3 × cellular IC50 corrected for free fraction) for target PK/PD (Figure 6). For the same coverage, the SC dose of the nanosuspension enabled a reduced total dose amount and frequency. This provides a welcomed advantage for a chronic dosing setting where a reduced burden to animals and manpower are desired. In addition, the significantly reduced plasma P/T ratio is less confounding for the interpretation of PK/PD relationships. When dosed s.i.d. via SC, a DCU plasma P/T ratio of 4 is expected (compare b.i.d oral P/T ratio of 25). When dosed b.i.d. via SC, a DCU plasma P/T ratio of less than 2 is expected (compare to t.i.d oral P/T ratio of >8). Our investigation with DCU provides an example of how nanosuspension can serve as a powerful formulation for the delivery of low solubility compounds in the preclinical setting. Based upon the positive results of our investigation, the continued use of nanosuspension to deliver low solubility compounds in preclinical PK/PD studies is expected.

## Conclusion

It is well-known that safety and efficacy are the two major concerns of any new therapeutic target. Failure to fully understand either target safety or efficacy in the early development process often results in a more costly failure later in the clinic or even postmarketing. For this reason, the pharmaceutical industry spends significant resources on early target evaluation in order to minimize the risk in moving forward. However, such a process often relies on finding a suitable compound to interrogate the target which may take a considerable time and is not cost effective. Here, we describe an effort with a less than ideal model compound, DCU, utilizing nanosuspension formulation and careful evaluation using PK modeling and simulation. This approach helped us identify clear advantages of using nanosuspension. In addition, we were able to evaluate SC delivery of DCU which has distinct advantages when compared to what has been previously described in literature. We firmly believed that using the systematic approach will enable earlier "preclinical proof of concept studies" and ultimately save both time and resources when investigating new and novel targets. Further research is needed to continue the development in this area.

## Competing interests

The authors declare that they have no competing interests.

## Authors' contributions

PCC conceived, design, and coordination the study. YR, KJC, and YC contributed to the manuscript. HW carried out the animal study.

## References

[B1] Katoh T Takahashi K Capdevila J Karara A Falck J Jacobson H Badr K Glomerular stereospecific synthesis and hemodynamic actions of 8,9-epoxyeicosatrienoic acid in rat kidney Am J Physiol 1991 261 578 586 10.1152/ajprenal.1991.261.4.F578 1928373

[B2] Lin W Falck J Wong P Effect of 14,15-epoxyeicosatrienoic acid infusion on blood pressure in normal and hypertensive rats Biochem Biophys Res Commun 1990 167 977 981 10.1016/0006-291X(90)90619-X 2322287

[B3] Imig J Navar L Roman R Reddy K Falck J Actions of epoxygenase metabolites on the preglomerular vasculature J Am Soc Nephrol 1996 7 2364 2370 8959626 10.1681/ASN.V7112364

[B4] Node K Huo Y Ruan X Yang B Spiecker M Ley K Zeldin D Liao J Anti-inflammatory properties of cytochrome P450 epoxygenase-derived eicosanoids Science 1999 285 1276 1279 10.1126/science.285.5431.1276 10455056 PMC2720027

[B5] Roman R P-450 metabolites of arachidonic acid in the control of cardiovascular function Physiol Rev 2002 82 131 185 11773611 10.1152/physrev.00021.2001

[B6] Spector A Fang X Snyder G Weintraub N Epoxyeicosatrienoic acids (EETs): metabolism and biochemical function Prog Lipid Res 2004 43 55 90 10.1016/S0163-7827(03)00049-3 14636671

[B7] Imig J Epoxide hydrolase and epoxygenase metabolites as therapeutic targets for renal diseases Am J Physiol Renal Physiol 2005 289 F496 F503 10.1152/ajprenal.00350.2004 16093425

[B8] Zhao X Imig J Kidney CYP450 enzymes: biological actions beyond drug metabolism Current Drug Metabolism 2003 4 73 84 10.2174/1389200033336892 12570747

[B9] Yu Z Xu F Huse L Morisseau C Draper A Newman J Parker C Graham L Engler M Hammock B Zeldin D Kroetz D Soluble epoxide hydrolase regulates hydrolysis of vasoactive epoxyeicosatrienoic acids Circ Res 2000 87 992 998 11090543 10.1161/01.res.87.11.992

[B10] Davis B David A Howard L Morisseau C Hammock B Weiss R Inhibitors of soluble epoxide hydrolase attenuate vascular smooth muscle cell proliferation PNAS 2002 99 2222 2227 10.1073/pnas.261710799 11842228 PMC122346

[B11] Ross R The pathogenesis of atherosclerosis: a perspective for the 1990s Nature (London) 1993 362 801 809 10.1038/362801a0 8479518

[B12] Smith K Pinkerton K Watanabe T Pedersen T Ma S Hammock B Attenuation of tobacco smoke-induced lung inflammation by treatment with a soluble epoxide hydrolase inhibitor PNAS 2005 102 2186 2191 10.1073/pnas.0409591102 15684051 PMC548576

[B13] Chiang P Wahlstrom J Selbo J Zhou S Wene S Albin L Warren C Smith M Roberds S Ghosh S Zhang L Pretzer D 1,3-Dicyclohexyl urea nanosuspension for intravenous steady-state delivery in rats J of Exp Nano 2006 2 239 250

[B14] Ghosh S Chiang P Wahlstrom J Fujiwara H Selbo J Roberds S Oral delivery of 1,3-dicyclohexylurea nanosuspension enhances exposure and lowers blood pressure in hypertensive rats Basic Clin Pharmacol Toxicol 2008 102 5 453 458 10.1111/j.1742-7843.2008.00213.x 18312493

[B15] Wahlstrom J Chiang P Ghosh S Warren C Wene S Albin L Smith M Roberds S Pharmacokinetic evaluation of a 1,3-dicyclohexylureananosuspension formulation to support early efficacy assessment Nanoscale Res Lett 2006 2 291 296

[B16] Bittner B Mountfield R Intravenous administration of poorly soluble new drug entities in early drug discovery: the potential impact of formulation on pharmacokinetic parameters Curr Op Drug Discov Devel 2002 5 1 59 71 11865674

[B17] Barrett R Nanosuspensions in drug delivery Nat Rev Drug Discov 2004 3 785 796 10.1038/nrd1494 15340388

[B18] Chiang P Hu Y Thurston A Sommers C Guzova J Kahn L Lai Y Blom J Pharmacokinetic and pharmacodynamic evaluation of the suitability of using fluticasone and an acute rat lung inflammation model to differentiate lung versus systemic efficacy J Pharm Sci 2009 98 11 4354 4364 10.1002/jps.21714 19230021

[B19] Liu C Research and Development of Nanopharmaceuticals in China Nano Biomed Eng 2009 1 1 1 18

[B20] Liu Y Miyoshi H Nakamura M Nanomedicine for drug delivery and imaging: a promising avenue for cancer therapy and diagnosis using targeted functional nanoparticles Int J Cancer 2007 120 12 2527 2537 10.1002/ijc.22709 17390371

[B21] Urisu T Wei C America--Japan Nanomedicine Society (AJNS) Nanomedicine 2006 2 4 297 298

[B22] Liang X Chen C Zhao Y Jia L Wang P Biopharmaceutics and therapeutic potential of engineered nanomaterials Curr Drug Meta 2008 9 8 697 709 10.2174/138920008786049230 PMC2715162 18855608

[B23] Si D Sun Y Cheng T Liu C Biomedical evaluation of nanomedicines Asian Journal of Pharmacodynamics and Pharmacokinetics 2007 7 2 83 97

[B24] Badyl D Lata H Dadhich A Animal models of hypertension and effect of drugs Indian J Pharmacol 2003 35 349 362

[B25] Lai Y Chiang P Li N Shevlin K Brayman T Hu Y Selbo J Hu L Comparison of in vitro nanoparticles uptake in various cell lines and in vivo pulmonary cellular transport in intratracheally dosed rat model Nonoscale Res Lett 2008 3 321 329 10.1007/s11671-008-9160-2

[B26] Chiang P South S Wene S The impact of dosing interval in a novel tandem oral dosing strategy: enhancing the exposure of low solubility drug candidates in a pre-clinical setting J Drug Delivery 2011 Article ID 528284, 9 pages. 10.1155/2011/528284 PMC3065744 21490753

[B27] Chiang PC South SA Foster KA Daniels JS Wene SP Albin LA Thompson DC Utilizing a novel tandem oral dosing strategy to enhance exposure of low solubility drug candidates in pre-clinical setting J Pharm Sci 2010 99 7 3132 3140 20229600 10.1002/jps.22092

[B28] Oberle R Chen T Lloyd C Barnett J Owyang C Meyer J Amidon G The influence of the interdigestive migrating myoelectric complex on the gastric emptying of liquids Gastroenterology 1990 99 5 1275 1282 2210236 10.1016/0016-5085(90)91150-5

[B29] Chiang P Kishore N Thompson D Combined use of pharmacokinetic modeling and a steady state delivery approach allow early assessment of IκB kinase-2 (IKK-2) target safety and efficacy J Pharm Sci 2010 99 3 1278 1287 19743500 10.1002/jps.21909

[B30] Kerns E Di L Drug-like properties: concepts, structure design and methods: from ADME to toxicity optimization 2008 Burlington: Elseveir

